# Catalytic reduction of toxic dyes over nickel oxide nanoparticles supported on CMK-3 catalyst

**DOI:** 10.1038/s41598-024-66243-2

**Published:** 2024-07-17

**Authors:** Mohammed M. Younus, M. A. Sayed, Mohamed El Saied, Ahmed O. Abo El Naga

**Affiliations:** 1 Special Processes Lab, Processes Development Division, Egyptian Petroleum Research Institute, EPRI, Nasr City, Cairo, 11727 Egypt; 2 Refining Division, Egyptian Petroleum Research Institute, Nasr City, Cairo, 11727 Egypt

**Keywords:** CMK-3, Methylene blue, Methyl orange, Reduction of organic dyes, Environmental sciences, Chemistry, Materials science

## Abstract

In the current paper, a NiO nanoparticles-loaded mesoporous carbon (CMK*-*3) catalyst, denoted as NiO/CMK-3, has been successfully synthesized using a facile strategy. The as-prepared material has been characterized through XRD, Raman spectroscopy, low-temperature N_2_ physisorption measurements, FTIR, FE-SEM, TEM, and XPS. The as-fabricated NiO/CMK-3 catalyst manifested a superior activity in the NaBH_4_-assisted reduction of methylene blue (MB) dye to its colorless leuco form. Remarkably, over 99% of 25 mg L^−1^ MB was reduced by 7.5 mM/L NaBH_4_ using 0.1 g L^−1^ NiO/CMK-3 within 3 min at room temperature. Furthermore, the kinetics study confirmed the appropriateness of the pseudo-first-order kinetic model for elucidating the kinetics of MB reduction by the catalyst. Importantly, the NiO/CMK-3 catalyst maintained almost constant catalytic activity even after 5 times of reuse in MB reduction, demonstrating its superior stability and reusable ability. So, NiO/CMK-5 appears as a promising heterogeneous catalyst for the effective remediation of dye-containing wastewater.

## Introduction

Many industries, such as textile, plastics, paper, and pulp, generate streams of waste effluents that contain considerable amounts of organic dyes. Sauer et al.^1^ reported that approximately 1–15% of the world's total production of dyes is lost in wastewater during the dyeing process. When these compounds are discharged into the main water bodies without prior treatment, they can cause an ecological imbalance. These molecules have carcinogenic and mutagenic properties towards aquatic organisms, posing a threat to human life at the end of the food chain^2–4^. In addition, the contamination of wastewater with these dyes depletes dissolved oxygen, affecting aquatic life, and causing environmental problems^3^.

So far, numerous processes have been proposed to remediate dye-contaminated effluents from industrial processes, including biodegradation^5^, chemical oxidation^6^, chemical reduction^7^, flocculation^8^, adsorption^2^, and photocatalysis^9^. Lately, the chemical reduction strategy using NaBH_4_ in the presence of suitable catalysts has gained more and more research attention^7^. Superior performance in removing several highly poisonous and persistent organic dyes with milder reaction conditions, convenient operation, cost-effectiveness, and low energy consumption are the paramount benefits of this strategy^7,10,11^. Nevertheless, so far, the vast prevalence of the effective heterogeneous catalysts documented in the literature for NaBH_4_-assisted reduction of organic dyes are based on expensive and scarce precious metal nanoparticles^12,13^. Accordingly, fabricating inexpensive and stable heterogeneous catalysts that can efficiently catalyze this process under ambient circumstances is of paramount interest.

Nickel oxide (NiO) nanoparticles, as one of the most important transition metal oxides, have garnered immense research and technological interest in the past decades^14^. This high importance is attributed to their intriguing physical and chemical properties^14^, as well as their widespread potential use in a variety of technologically vital fields, including gas sensors^15^, energy storage devices^16^ and batteries^17^, electrical conductivity^18^, magnetism^19^, and heterogeneous catalysis^20^. Due to its extraordinary electrical, thermal, and redox features, NiO has been recognized as advantageous nanocatalysts in diverse catalytic reactions^20^, such as oxygen evolution reaction catalysis^21^, catalytic ozonation^20^, CO_2_ methanation^22^, biomass Gasification^23^, methane oxidation^24^, water splitting^25^, hydrogenation of levulinic acid^26^, among others. Unfortunately, the inevitable severe aggregation/agglomeration of metal nanoparticles (MNPs) is commonly noticed throughout the course of liquid-phase reactions, leading to a significant loss of activity^27^. A plausible route to overcome the challenge of MNPs aggregation might be to incorporate them into appropriate high surface area solid support materials^28^. The incorporation of will enhance the exposure of the catalytically active centers, leading to a more efficient catalytic process^29^. Notably, the metal–support interaction leads to a firm fixation of the MNPs to the catalyst surface, deterring their aggregation and giving rise to a catalyst with long-term stability during the catalytic process^30^.

Ordered mesoporous carbons (OMCs) represent a captivating category of carbon materials vastly employed as supporting materials for diverse MNPs in executing a variety of chemical reactions^31,32^. Among various OMCs, CMK-3 is the most scrutinized for this purpose in view of its notable features, including large specific surface area, large pore volume, adaptable pore sizes, interconnected porous network, as well as excellent thermal and chemical stabilities^33–35^. CMK-3 is conventionally fabricated by the nanocasting approach, adopting mesoporous SBA**-**15 as the hard template and glucose or sucrose as the carbon source.

In the present work, NiO-loaded CMK-3 (referred to as NiO/CMK-5) has been synthesized as a superior heterogeneous catalyst for the NaBH_4_-assisted reductive decolorization of toxic organic dyes at room temperature. The NiO/CMK-5 catalyst demonstrates a high normalized first-order reaction rate constant of 3.5 × 10^−2^ s^−1^, an MB decolorization degree of 99.2% within 3 min, and superior reusability for five successive reduction rounds. Therefore, NiO/CMK-5 appears as a promising heterogeneous catalyst for the remediation of dye-containing wastewater.

## Experimental

### Preparation of CMK-3

CMK-3 was prepared using the methods described in the literatures^36^.

### Synthesis of nickel oxide nanoparticles

The solution combustion synthesis (SCS) approach was used to fabricate nickel oxide nanoparticles, utilizing Ni(NO_3_)_2_⋅6H_2_O as the nickel source and urea as the fuel^37^. Firstly, a specific quantity of Ni(NO_3_)_2_⋅6H_2_O, (3.7 g) and urea (1.3 g) was dissolved in 50 mL of de-ionized water. Then, the resulting solution was allowed to stir for 30 min at room temperature, before being ignited in a muffle furnace at 400 °C. Finally, the obtained green solid was calcinated at a muffle furnace at 550 °C for 3 h.

### Synthesis of NiO/CMK-3 catalyst

The NiO/CMK-5, catalyst with a NiO loading of 5 wt%, was synthesized as follows: 1.0 g of as-obtained CMK-3 was placed in 50 mL of absolute EtOH and sonicated for 1 h. Subsequently, the desired quantity of the as-obtained NiO powder was gradually added to the CMK-3 dispersion under sonication. The resulting suspension was permitted to stir at ambient temperature for 12 h before being evaporated to dryness at 80 °C for 5 h. The resulting powder was then oven-dried at 100 °C overnight and ultimately calcined in flowing nitrogen at 500 °C for a period of 2 h. The final solid catalyst was referred to as NiO/CMK-3.

### Catalyst characterization

To evaluate porous properties of the catalyst, N_2_-adsorption/desorption isotherms at 77 K were recorded using a Quanta Chrome Nova 3200 analyzer. A Nexus-870 FTIR spectrometer was employed to record the Fourier transform infrared (FTIR) spectra of the obtained materials. Raman spectroscopy was conducted at room temperature using SENTERRA Dispersive Raman Microscope. X-ray photoelectron spectroscopy (XPS) was utilized to identify the catalyst structure with a Thermo Scientific X-ray photoelectron spectrometer. Powder X-ray diffraction patterns were recorded using a X'Pert PRO MPD PANalytical diffractometer at the 2θ range from 10° to 90°.

### Catalytic activity tests

The NaBH_4_-assisted reduction experiments were executed at 25 ℃ in a 500 mL glass beaker containing 200 mL of dye at the desired concentration under continuous mechanical stirring. In a typical experiment, 0.025 g catalyst was introduced into the dye solution, followed by the addition of NaBH_4_ at 7.5 mM/L. After specific time intervals elapsed, samples were withdrawn from the reaction mixture, filtered via a syringe filter to get rid of the suspended catalyst, and analyzed for their remaining dye concentration via a UV–vis spectrophotometer (JASCO, V-750 UV–visible Spectrophotometer). Each reduction experiment was conducted three times, and the mean values were used.

## Results and discussions

### Catalyst characterization

**Figure 1:**
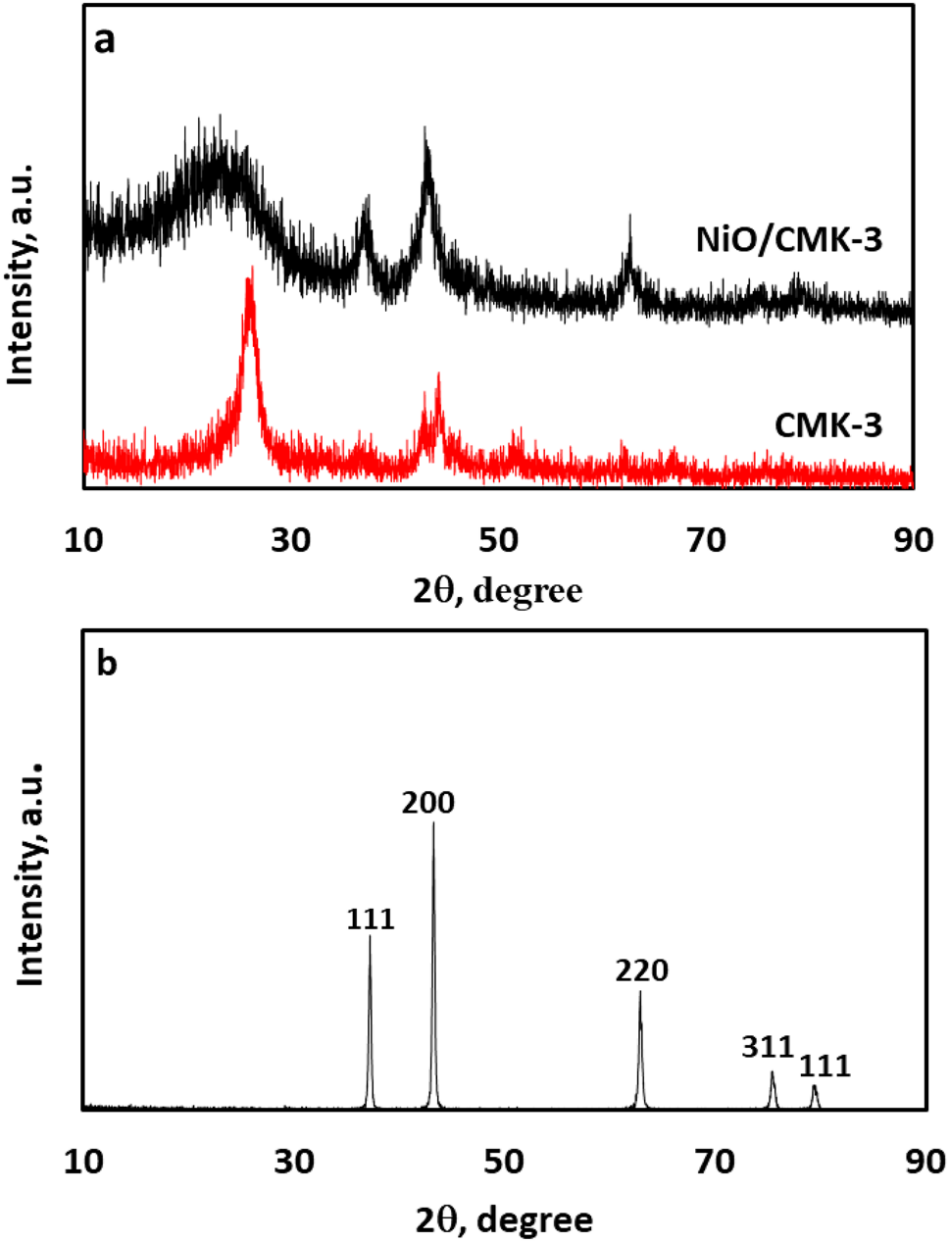
(**a**) XRD patterns of the CMK-3 and NiO/CMK-3, and (**b**) NiO nanoparticles.

The XRD diffractograms of CMK-3 and the NiO-loaded catalyst are illustrated in Fig. 1a. For the sake of comparison, the diffractogram of NiO nanoparticles is also presented in Fig. 1b. As for the pure CMK-3 material, two broad reflections can be observed at 2θ values of about 24° and 44°, corresponding to the (110) and (200) diffraction facets of graphite, respectively, revealing the low crystalline extent of the as-obtained CMK-3^29^. Regarding the XRD diffractogram of the NiO/CMK-3 catalyst, the reflections positioned at 2θ values of approximately 37.2°, 43.29, 62.85, 75.35, and 79.15 originated from the (111), (200), (220), (311), and (11) diffraction facets of NiO (JCPDS File No. 78-0643), respectively. This affirms that the NiO species has been successfully loaded. Please note that the wideness of the reflections attributed to NiO reveals the small size of the NiO nanoparticles in the composite^38^. The average crystallite size of the NiO nanoparticles was estimated to be approximately 9.87 nm based on the value of full width at half maximum (FWHM) of the (200) diffraction peak, adopting the Debye–Scherrer equation.

**Figure 2:**
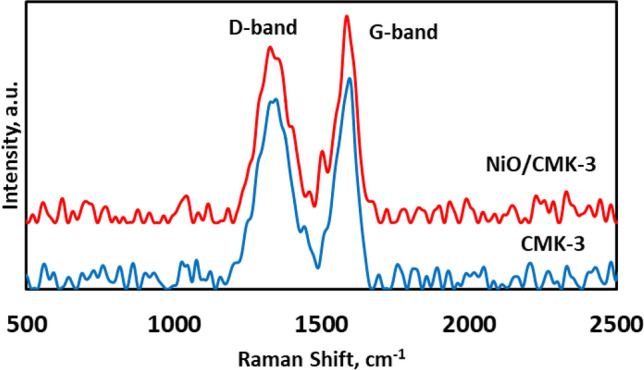
Raman spectroscopy of CMK-3 and NiO/CMK-3.

Figure 2 illustrates the Raman spectra of the CMK-3 and NiO/CMK-3 samples. Considering the bare CMK-3, two prominent peaks located at wavenumbers of about 1310 cm^−1^ and 1660 cm^−1^ are evident, corresponding to the disordered (D-band) and graphitic (G-band) carbon structure, respectively^39–41^. The ID**/**IG area ratio of the CMK-3 was computed to be 0.99, revealing its low graphitization extent, in good accord with the results from the XRD study. The NiO/CMK-3 hybrid demonstrates an almost similar Raman spectrum to CMK-3, signifying that the NiO loading does not influence the structure of the pure support material.

**Figure 3:**
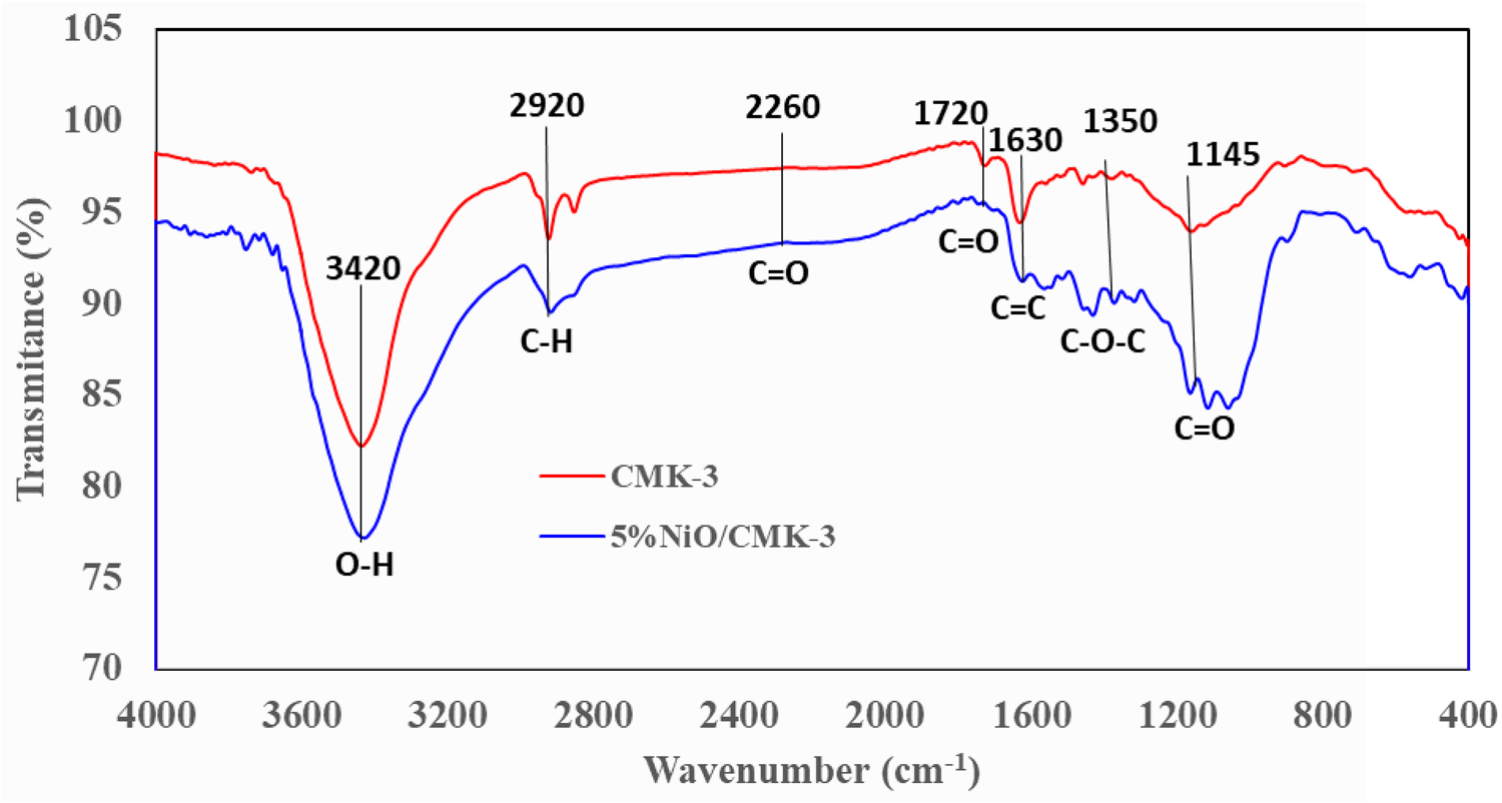
FT-IR spectra of the CMK-3 along with NiO/CMK-3.

FTIR analysis was executed to scrutinize the chemical nature of the NiO/CMK-3 catalyst. FT-IR spectra of CMK-3 and NiO/CMK-3 are depicted in Fig. 3. The FTIR spectrum of the pristine CMK-3 depicts an absorption band at around 1133.5 cm^−1^, which is attributed to the stretching modes of the aromatic C–O bonds^39^. The absorption peak assigned to the C=C stretching vibrations was obviously observable at approximately 1619 cm^−1^^41^. In addition, the band emerging at 3404 cm^−1^ belongs to the O–H stretching vibrations^42,43^, whereas the peak at 2916 cm^−1^ is likely to arise from methylic vibration^39^. All these characteristic peaks distinctly insinuate that the expected mesoporous carbon material was successfully synthesized employing the adopted synthetic strategy. An identical spectrogram was observed for the NiO-loaded catalyst, revealing that the structure of the support material remained basically unaltered after the loading of NiO nanoparticles.

**Figure 4:**
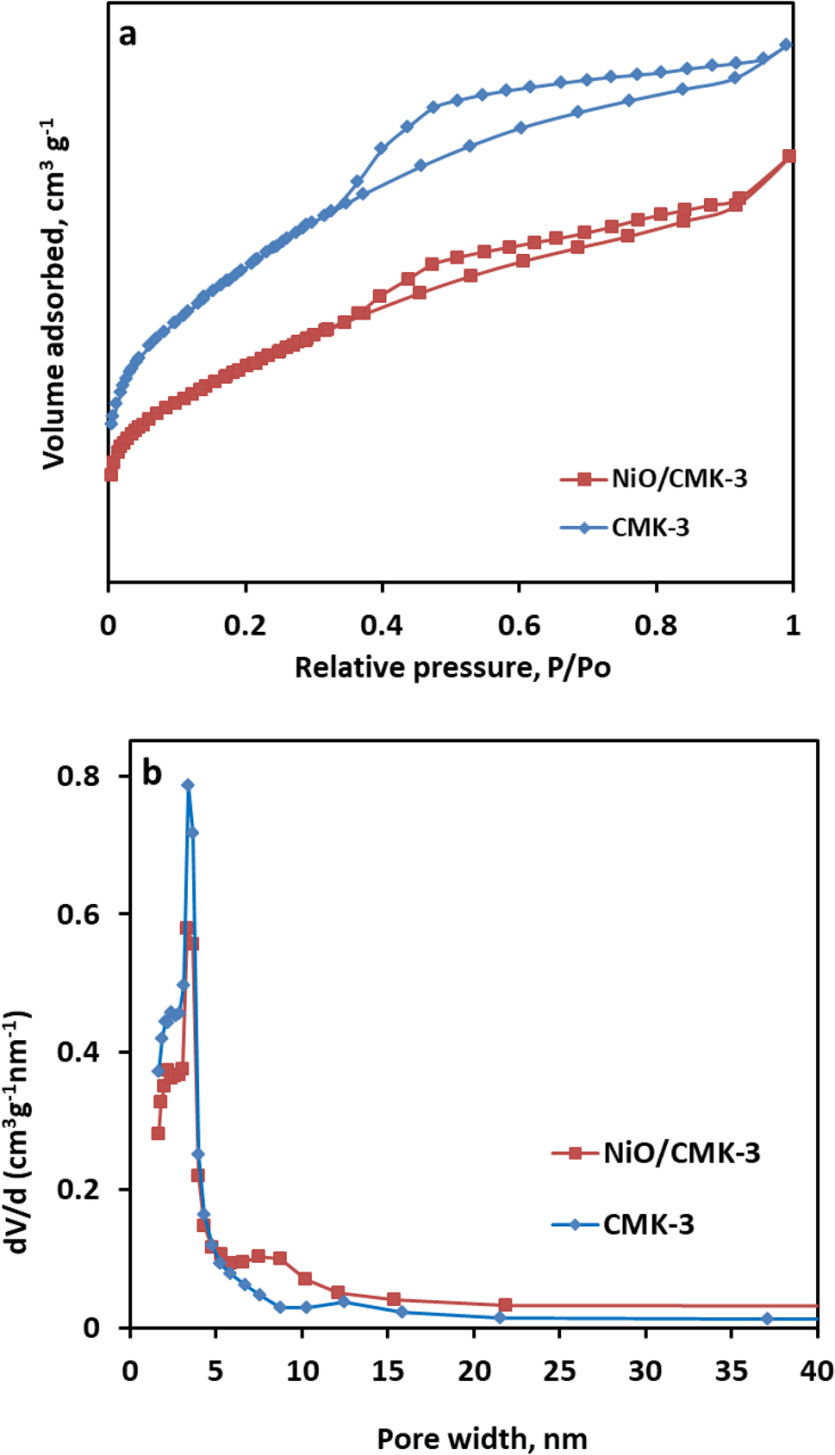
(**a**) N_2_ adsorption–desorption isotherms, (**b**) BJH pore size distribution plots of CMK-3 and NiO/CMK-3.

The porous attributes of the synthesized materials were dissected through low-temperature nitrogen physisorption experiments. The resulting N_2_ sorption isotherms and BJH pore size distribution plots are manifested in Fig. 4. As depicted in Fig. 4a, the isotherm of the CMK-3 material is identified as type-IV isotherms, typical for mesoporous materials with pore widths in the range from 2 to 50 nm. A pronounced hysteresis loop also appears at a relative pressure from 0.45 to 1.0, caused by the capillary condensation of N_2_ in mesopores. The NiO-containing material (Fig. 4a) retained the same isotherm shape, ascertaining that the original mesoporous system of the parent support material was well conserved after loading NiO species. Please note that NiO/CMK-3 adsorbed less nitrogen compared to bare CMK-3, and the hysteresis loop becomes less obvious, signifying the successful incorporation of the NiO nanoparticles inside the porous system of CMK-3. The BJH pore size distribution plots derived from the isotherms, Fig. 4b, further demonstrate the mesoporous features of the two samples.

CMK-3 has a BET-specific surface area and total pore volume of about 495 m^2^ g^−1^ and 0.37 cm^3^ g^−1^, respectively. As expected, the BET-specific surface area and total pore volume of the pristine CMK-3 obviously dropped from to 792.3 to 328.7 m^2^ g^−1^ and 0.58 to 0.31 cm^3^ g^–1^, respectively, after the loading of NiO species. This implies that the porous system of the CMK-3 is partially occupied by the NiO nanoparticles. It is worth mentioning that NiO/CMK-3 catalyst still possesses a relatively high surface area and pore volume, which are critical for effective catalytic process.

**Figure 5:**
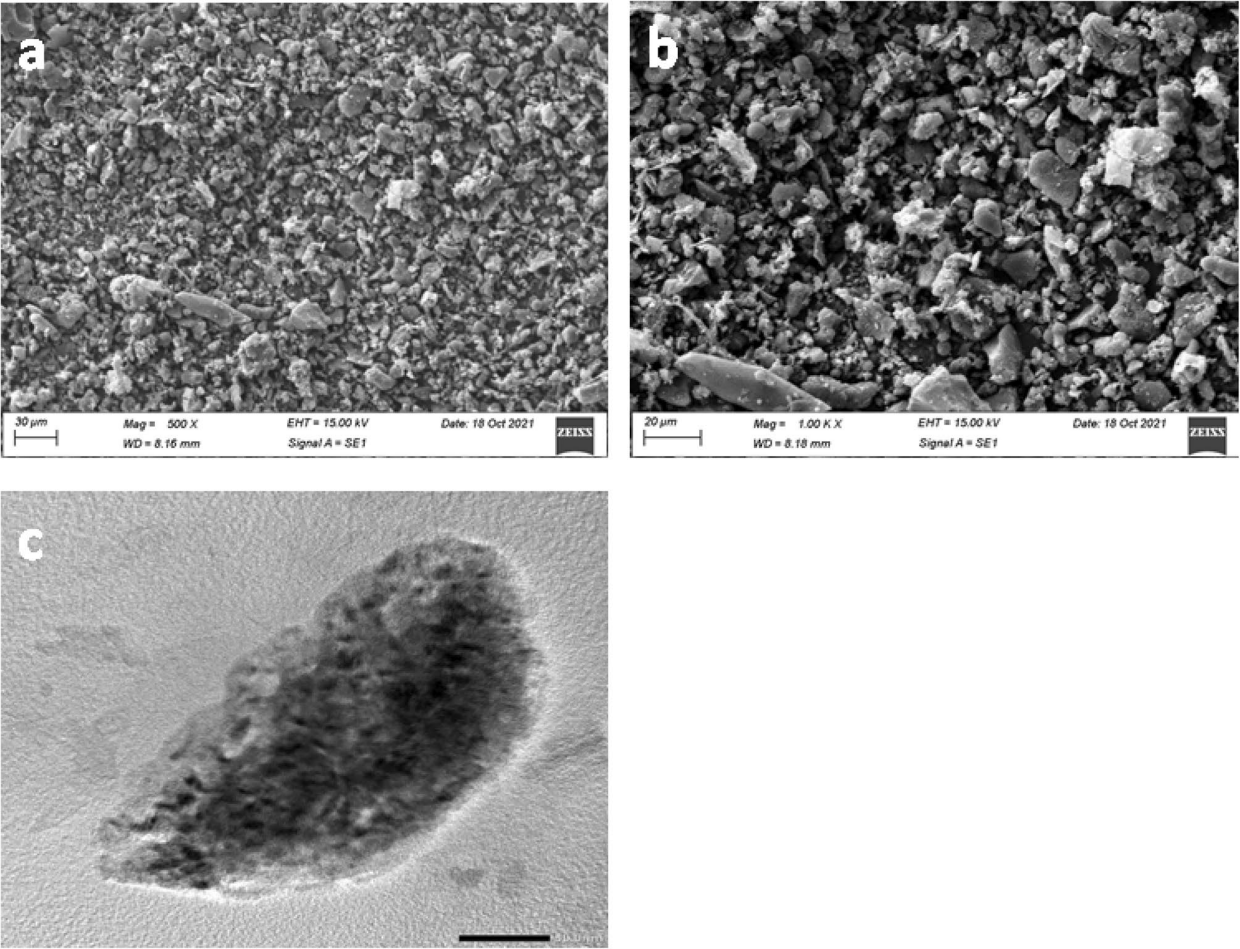
(**a**,**b**) SEM, and (**c**) TEM images of NiO_3_/CMK-3 nanocomposite.

The morphologies of the synthesized NiO/CMK-3 catalyst were investigated through scanning electron microscopy (SEM) and transmission electron microscopy (TEM) techniques. The SEM images, in Fig. 5a,b, shows highly agglomerated nanoparticles with irregular shape and rough surfaces. The TEM image reveals that NiO NPs are uniformly distributed in the CMK nano layer with diameters ranging from 6 to 10 nm (Fig. 5c).

**Figure 6:**
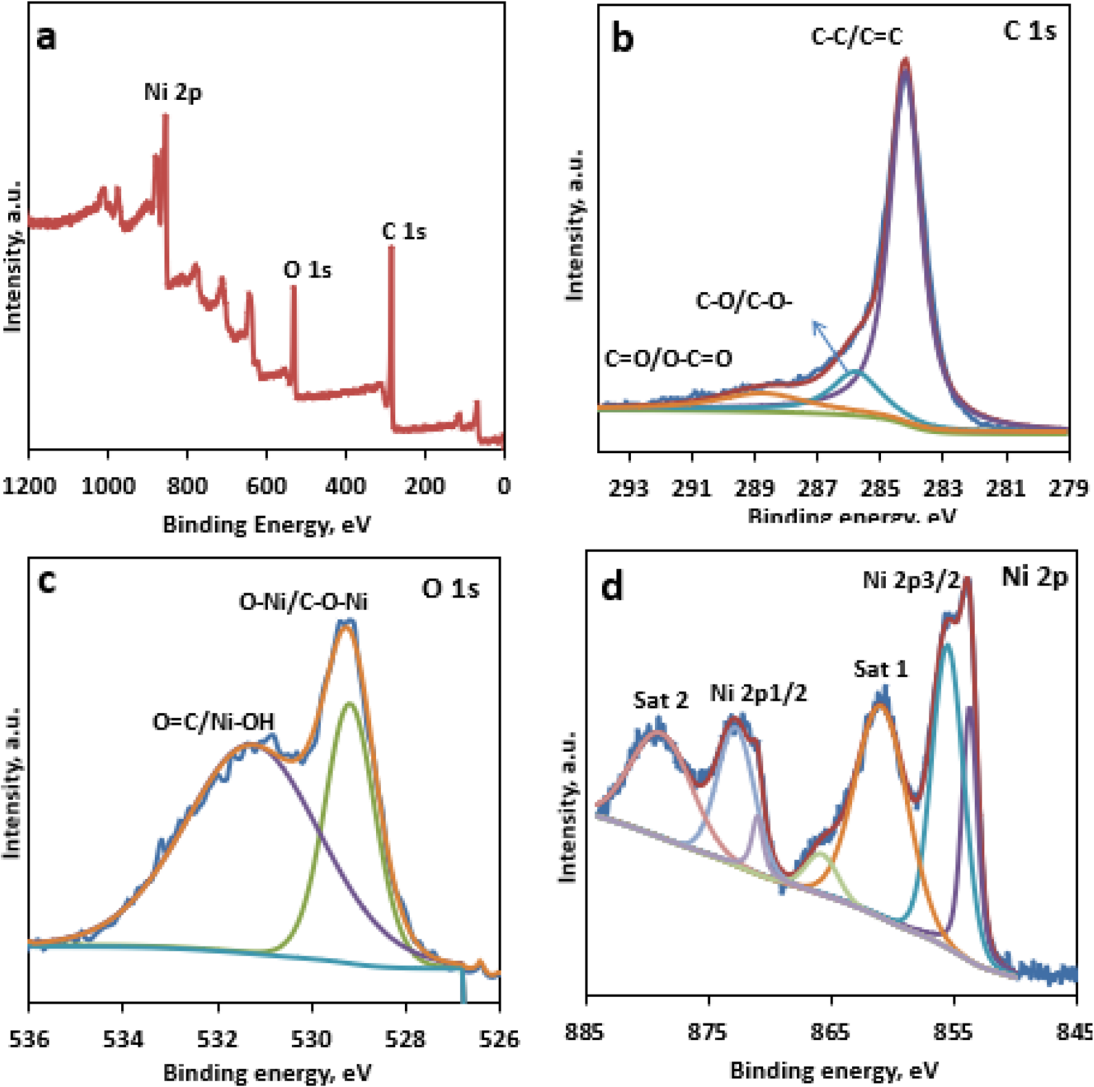
XPS spectra of NiO/CMK-3 catalyst: (**a**) survey scan, (**b**) C 1s, (**c**) O 1s, and (**d**) Ni 2p.

The XPS survey spectra of NiO@CMK-3 (Fig. 6a) confirmed the existence of C, O, and Ni elements. The chemical valence of the surface species in the NiO@CMK-3 catalyst was additionally assessed by XPS spectra. The XPS spectra of the C 1s peaks were deconvoluted into three spectral bands at 284.2 eV, 285.8 eV, and 288.8 eV. The prominent peak located at 284.2 eV was assigned to C in C–C/C=C, and the peaks at 285.8 eV and 288.8 eV corresponded to C in C–O/C–O–C and C in –C=O/O–C = O, respectively, (Fig. 6b)^44,45^. The prominence of the spectral peak at 484.2 eV means that CMK-3 is predominantly made up of C–C. The deconvolution of the XPS O 1s spectra (Fig. 6c) gives rise to two defined peaks located at 529.2 and 531.4 eV, which corresponded to Ni–O/C-O-Ni and Ni-OH/C=O, respectively^43^. In the deconvoluted Ni 2p spectra (Fig. 6d), the peaks that appeared at binding energies of 855.6 and 872.5 eV are related to Ni^2+^; meanwhile, Ni^3+^ is responsible for the small shoulder at 853.7 and 871.06 eV binding energy, revealing the existence of a small amount of Ni_2_O_3_ on the surface^46^.

### Catalytic reduction of toxic dyes

The catalytic performance of the as-obtained NiO/CMK-3 was scrutinized in the reductive decolorization of methylene blue (MB) dye at room temperature, adopting NaBH_4_ as an efficacious and eco-friendly reductant. MB is a cationic thiazine pigment that is vastly used for coloring and printing purposes in multifarious industries. Nonetheless, MB dye poses a grave peril to mankind's health and the eco**-**environment, so the effectual remediation of MB-containing industrial effluents is of massive importance^7^. The change of the decolorization degree of MB (represented by Ct/Co, where C_o_ (mg L^−1^) denotes the concentration of the MB solution before the commencement of the reduction reaction, and C_t_ (mg L^−1^) designates the remaining concentration of the dye solution after the elapse of time t of the reaction) during the process is drawn in Fig. 7. In the “NiO/CMK-3 + NaBH_4_” system, the decolorization degree of MB is accomplished within 3 min. For the sake of comparison, the reduction of MB (25 mg L^−1^) using the “NiO/CMK-3 only”, and “NaBH4 only” systems was also appraised (Fig. 7). For the “NiO/CMK-3 only” system, only slight decolorization degree of 32.1% was observed. Similarly, a**s** can be noticed from Fig. 7, the reduction of the MB dye essentially did not occur using the “NaBH4 only” system, which is likely due to the high activation energy barrier of the reaction^7^. The direct electron transfer from the electron donor (BH_4_^−^ anions) and the electron acceptor (MB molecules**)** is impeded as a result of the significant difference in redox potential between them. The NiO/CMK-3 catalyst functions as an electron mediator by accepting electrons from the BH_4_^−^ anions and conveying them to MB molecules. Such electron transfer disrupts the conjugated chromophoric system of MB, leading to its decolorization and the generation of the colorless Leucomethylene blue (LMB) product^7,47^.

**Figure﻿ 7:**
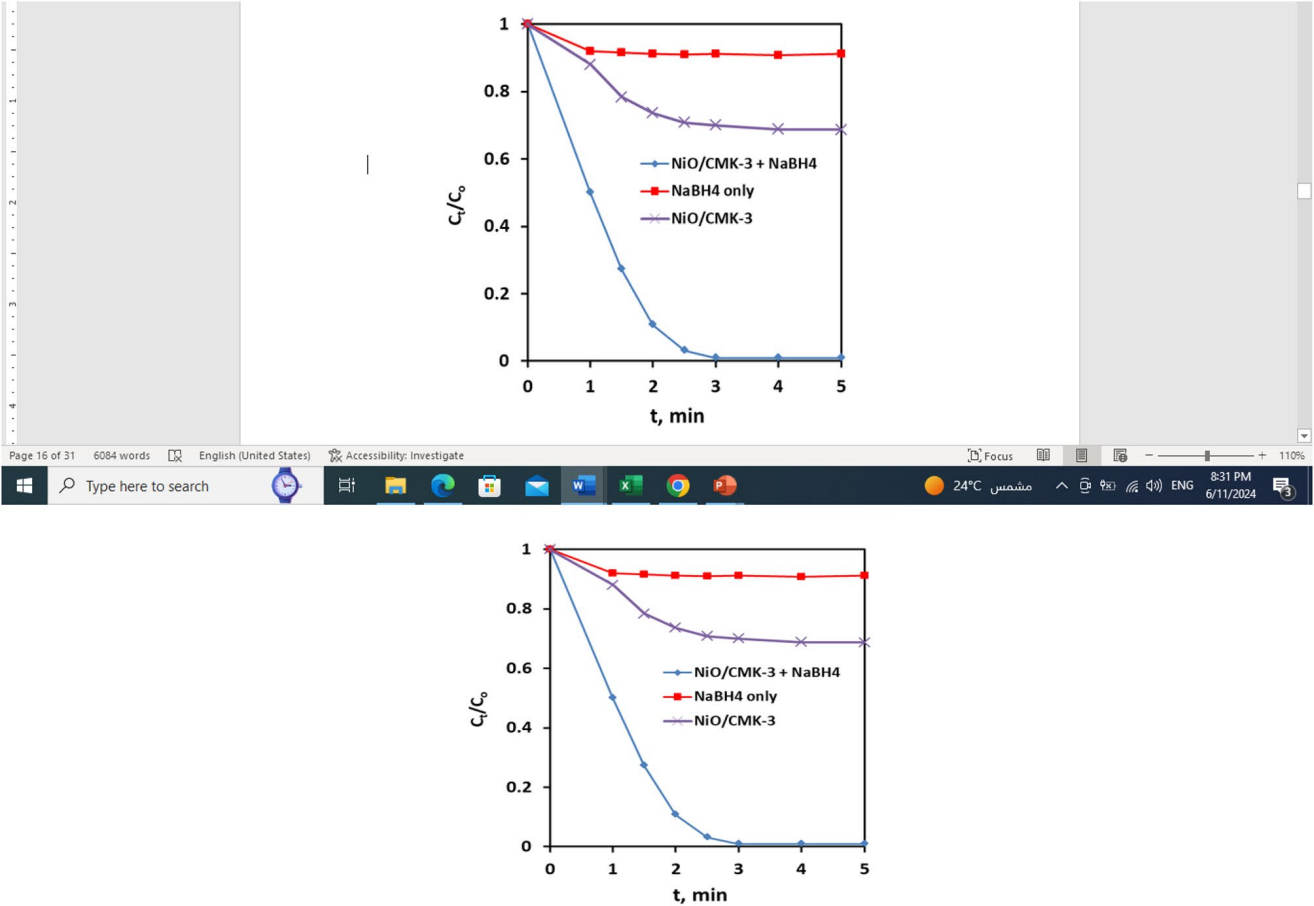
Reduction of MB by different systems.

#### Effect of catalyst dose on the catalytic reduction of MB

**Figure 8:**
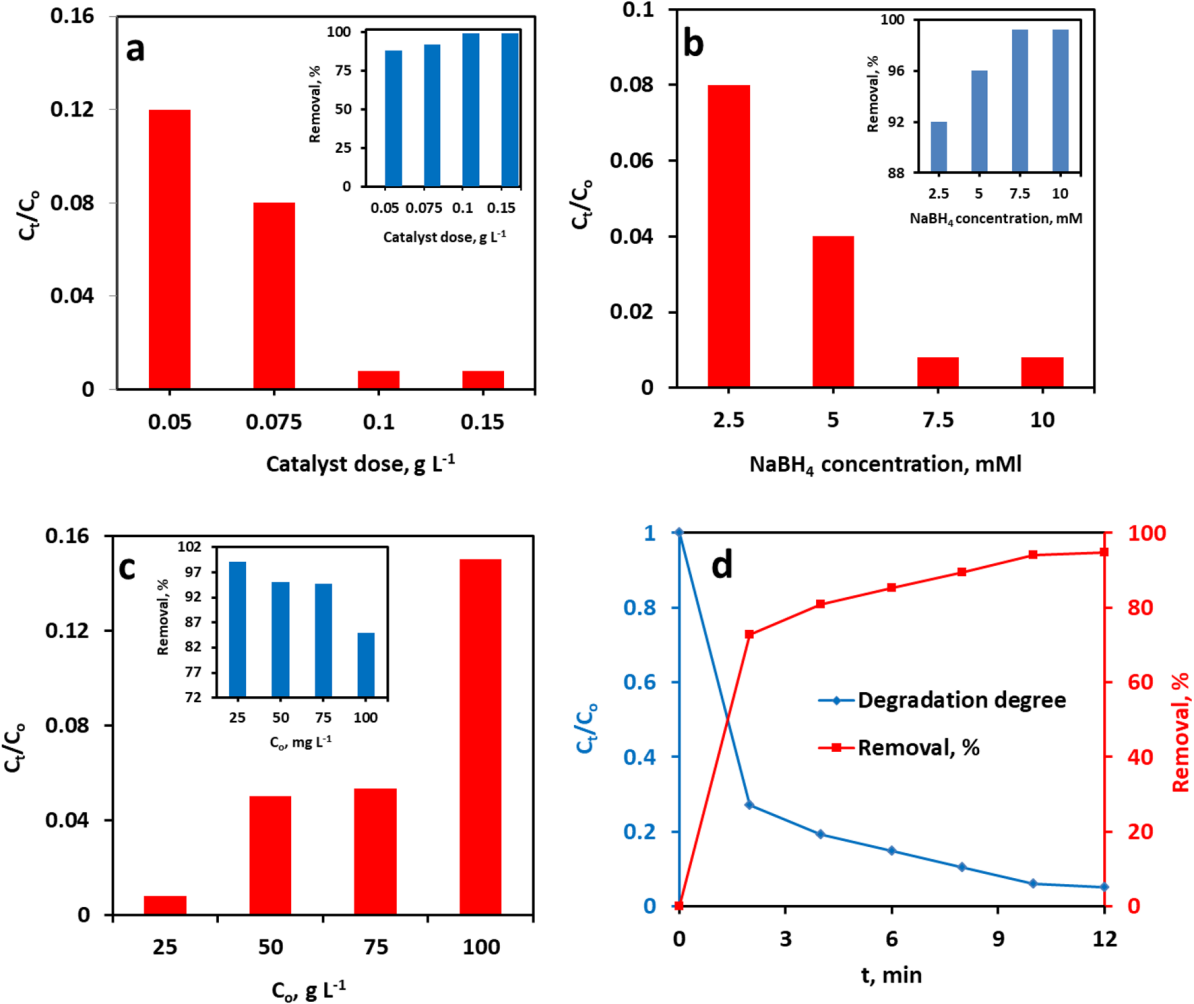
(**a**) Effect of catalyst dose on the decolorization degree with “NiO/CMK + NaBH4” system, (**b**) effect of NaBH_4_ concentration on the degradation rate with “NiO/CMK + NaBH4” system, (**c**) effect of MB initial concentration on the decolorization degree with “NiO/CMK + NaBH4” system, and (**d**) reduction of MO.

The ideal catalyst amount for the reduction of MB dye was determined by executing a series of reduction experiments with four different doses: 0.005, 0.075, 0.1, and 0.15 g L^−1^ of NiO/CMK-3. All other operational parameters were held unaltered: 25 °C, 3 min, [MB] = 25 mg L^−1^, [NaBH_4_] = 7.5 mM/L. The variation of the decolorization degree of MB as a function catalyst dose is plotted in Fig. 8a. Figure 8a depicts that the MB decolorization degree augmented as a function of NiO/CMK-3 dose, and the highest decolorization degree was attained at 0.1 g L^−1^, which is likely to arise from the increase in the number of available active centers for catalytic reductive decolorization of the MB dye^48^. Higher amounts of NiO/CMK-3 resulted in no further considerable improvement in the MB decolorization degree. Hence, 0.1 g L^−1^ of NiO/CMK-3 was taken as the optimum dose for maximum MB decolorization.

#### Effect of NaBH_4_ concentration on the catalytic reduction of MB

The impact of NaBH_4_ concentration on the decolorization degree of MB was further investigated. Figure 8b depicts the plot of decolorization degree MB versus NaBH4 concentration. For this purpose, different NaBH_4_ concentrations of 2.5, 5, 7.5 and 10 mM/L were used. All reduction tests were accomplished at 25 ℃ for 3 min, using 0.1 g L^−1^ NiO/CMK-3 dose. As perceived in Fig. 8b, with the concentration of NaBH_4_ increased from 2.5 to 7.5 mM/L, the dye decolorization degree augmented swiftly. The higher the concentration of NaBH_4_, the more BH_4_^-^ anions are created, which results in an increase in the electron concentration on the NiO/CMK-3 surface and the final decolorization degree of the MB dye^48^. Nonetheless, when the NaBH_4_ concentration surpassed 7.5 mM/L, there was no further notable increase in the degree of dye decolorization. In view of these findings, 7.5 mM/L of NaBH_4_ was chosen for the ongoing experiments.

#### Effect of dye concentration on the catalytic reduction of MB

The impact of increasing MB concentration in the range from 25 to 100 mg L^−1^ on the decolorization degree was further investigated, as illustrated in Fig. 8c. Other operational variables were kept unchanged at the following levels: temperature = 25 °C, time = 3 min, NaBH_4_ concentration = 7.5 mM/L, and catalyst dose = 0.10 g L^−1^. As expected, the decolorization degree of MB demonstrated a trend of continued lowering with the initial concentration. Raising MB concentration from 25 to 100 mg L^−1^; the MB removal efficiency is lowered from 99.2 to 85%. With the augmentation of MB concentration, the available catalytically active sites on the catalyst surface become insufficient, giving rise to a lower MB decolorization degree^48^. Please note that even in the presence of high concentrations of MB, a high decolorization degree can also be accomplished in a short reaction time (3 min), revealing that the NiO@CMK-3 was effective over a broad range of MB concentrations.

Besides, the NiO/CMK-3 catalyst underwent examination in the reductive degradation of methyl orange dye, MO, serving as a model of anionic dyes. This reaction was conducted adopting the optimized variables derived from the experiments on MB reduction (25 ºC, 0.1 g L^−1^ NiO/ CMK-3, and 7.5 mM/L NaBH_4_). As manifested in Fig. 8d, the degradation degree of MO dye reached 95.8% within 10 min. So, NiO/ CMK-3 catalyst proves to be an advantageous solid catalyst for the effective NaBH_4_-assisted reduction of both cationic and anionic dyes.

#### Kinetic study

Kinetic data derived from the NaBH4-assisted reduction of MB to LMB over NiO/CMK-3 was further scrutinized by adopting the pseudo-first-order kinetic model. (Eq. 1)^7^.

1 $$\text{ ln}({C}_{t}/{C}_{o})={k}_{app}t$$

where C_o_ (mg L^−1^) denotes the concentration of the MB solution before the commencement of the reduction reaction, C_t_ (mg L^−1^) designates the remaining concentration of the dye solution after the elapse of time t of reaction, and k_app_ (min^-1^) symbolizes the pseudo-first-order rate constant.

**Figure 9:**
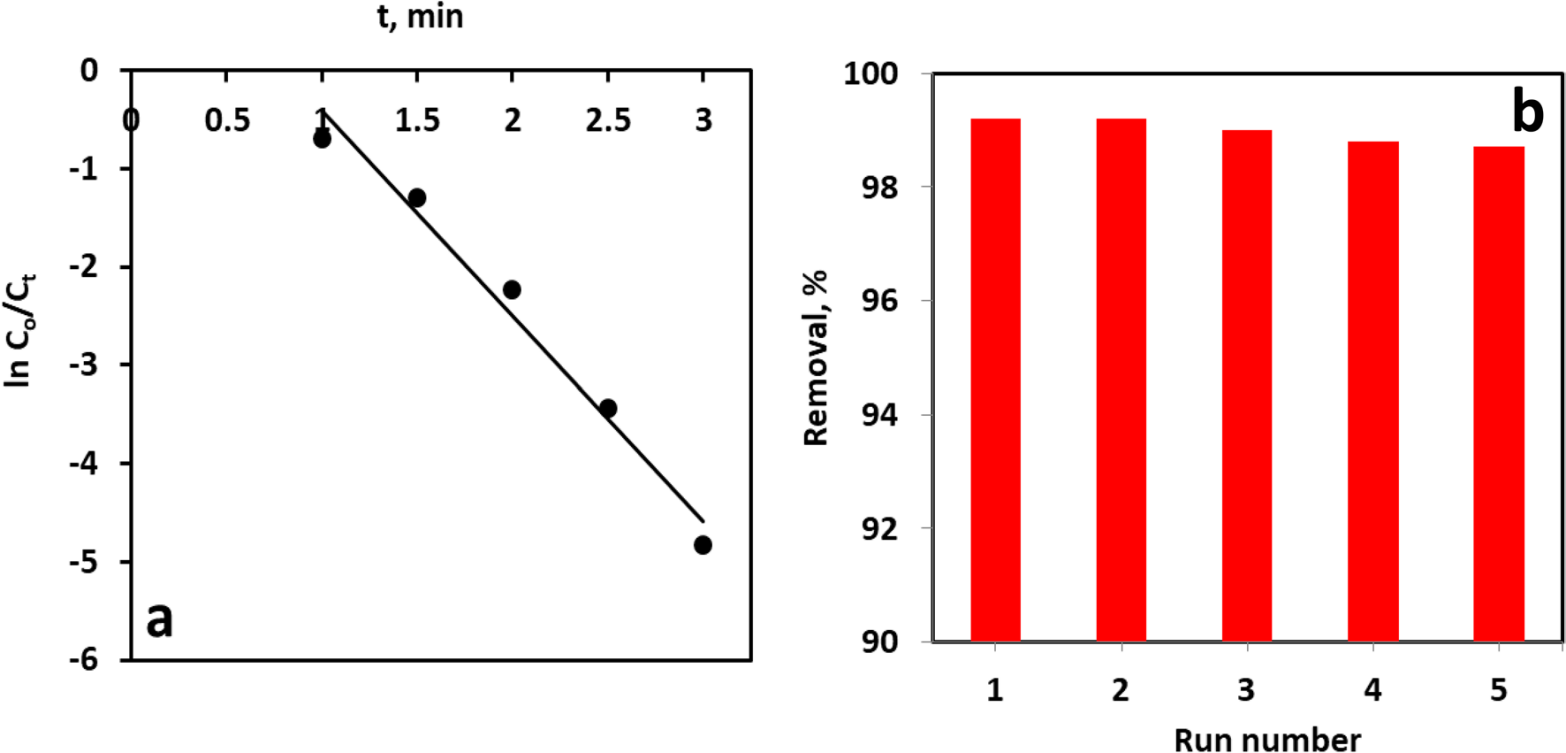
(**a**) Pseudo-first order kinetics for the reduction of MB by “NiO/CMK-3 + NaBH_4_” system, and (**b**) the recyclability of NiO/CMK-3 for five successive rounds of MB reduction.

As depicted in Fig. 9a, a perfect straight line was obtained when plotting against t, characterized by a high regression coefficient (R^2^) of 0.977. This underscores the appropriateness of the pseudo-first-order kinetic model in elucidating the mechanism of MB reduction by the catalyst. The perfect fit of this model insinuates that the reduction rate depends solely on the concentration of MB. From the slope of the line**,** the value of the apparent rate constant (*k*_app_) was calculated as 2.1 min^−1^.

In recent years, metal**-**based heterogeneous catalysts**`** have been intensively examined for the reduction of several organic dyes using NaBH_4_. A comparison of the reductive decolorization of MB by NaBH4 employing diverse heterogeneous catalysts is outlined in Table 1. Based on the value of the normalized first-order reaction rate constant, k_nor_, computed employing Eq. (2), the as-obtained NiO/CMK-3 material, subject to investigation in the current work, exhibits superior performance in the decolorization of MB when compared with the aforementioned catalysts. The outstanding performance of NiO/CMK-3 underscores its potential for applications in the remediation of dye-contaminated wastewater through the reductive degradation process.

2 $${k}_{nor}=\frac{{k}_{app}}{w}$$

where w denotes the mass of the NiO/CMK-3 catalyst.

**Table 1:** Comparison of some heterogeneous catalysts for the reduction of MB with NaBH_4_.

No.	Catalyst	C_o_, M	Mass of catalyst, mg	*k*_app_, S^−1^	*k*_nor_, s^−1^ mg^−1^	Ref.
1	CuS nanotubes	1.25 × 10^−4^	1	2.4 × 10^−2^	2.4 × 10^−2^	^7^
2	1.0PdAS	7.8 × 10^−5^	1	1 × 10^−2^	1 × 10^−2^	^49^
3	Fe_3_O_4_@PDA-Ag-10	1.2 × 10^−6^	10	7 × 10^−3^	7 × 10^−4^	^50^
4	3D-graphene/Ag	1 × 10^−3^	5	4.1 × 10^−3^	8.2 × 10^−4^	^51^
5	Au@TA-GH	3.1 × 10^−5^	2	5.1 × 10^−3^	2.5 × 10^−3^	^52^
6	Ni/CPM-1	3.0 × 10^−5^	2	9.51 × 10^−3^	4.8 × 10^−3^	^53^
7	NiO/CMK-3	7.8 × 10^−5^	1	3.5 × 10^−2^	3.5 × 10^−2^	This study

### Reusability

The stability and reusability of the catalyst in the reductive degradation of organic dyes are essential for the economic viability and environmental sustainability of the process. To assess the reusability of the NiO/CMK-3 catalyst, five cycles of the reduction reaction for MB were accomplished under identical process variables. After the completion of each reduction cycle, the used catalyst was isolated from the reaction mixture through centrifugation, rinsed copiously with distilled water, and oven-dried before being submitted to the next round of MB reduction. As depicted in Fig. 9b, the catalytic performance of the catalyst did not apparently drop after 5 times of reuse, demonstrating acceptable stability and reusable ability. Ni-leaching was also examined after the reduction reaction*,* unveiling only 0.1 mg/L of Ni in the solution after the catalytic cycle*.* This concentration is significantly below the maximum acceptable concentration of Ni in wastewater resources (900 mg/L)^54^. So, the weight loss of the catalyst during the recovery and washing processes can account for the observed slight drop in the decolorization degree upon repetitious usage. Furthermore, the used catalyst was characterized by FTIR to check the stability of the catalyst after the catalytic reduction of MB. The FT-IR spectra (see Fig. S1) of the NiO/CMK-3 was similar to that of the fresh sample, confirming that the structural integrity of the catalyst was well maintained after the catalytic run.

## Conclusions

In this study, a NiO/CMK-3 catalyst has been fabricated and employed for the NaBH4-assisted reduction of methylene blue dye. The catalyst manifested exceptional activity toward the reduction of methylene blue in the presence of NaBH4, achieving almost complete decolorization within 3 min at room temperature. Moreover, the results from the kinetic study unveiled the appropriateness of the pseudo-first-order kinetics to define the reductive decolorization of MB dye, with a high normalized rate constant of 3.5 × 10^−2^ s^−1^ mg^−1^. The catalyst was also utilized for the reductive degradation of MO dye, as a representative of anionic azo dyes, and the degradation degree reached 95.8% within 10 min. This establishes NiO/CMK-3 as an advantageous solid catalyst for the effective NaBH_4_-assisted reduction of both cationic and anionic dyes. Interestingly, the NiO/CMK-3 catalyst can be successfully recycled for five times for MB reduction with no pronounced lowering in the decolorization degree. In light of its superior catalytic performance, exceptional reusability, and efficacy in treating both cationic and anionic dyes, NiO/CMK-3 emerges as a promising catalyst for the highly efficient NaBH_4_-assisted reduction of toxic dyes in contaminated industrial effluents.

### Supplementary Information

Supplementary Figure S1.

## Data Availability

All data generated or analyzed during this study are included in this published article.
