# Supplementary material for: Catalytic reduction of toxic dyes over nickel oxide nanoparticles supported on CMK-3 catalyst
Source: Sci Rep. 2024 Jul 17;14:16583. doi: 10.1038/s41598-024-66243-2 (PMC11255306; doi:10.1038/s41598-024-66243-2)
Supplement: Supplementary file 1 — Supplementary Figure S1. [file 41598_2024_66243_MOESM1_ESM.docx]

Fig. S1. FT-IR spectra of the used NiO/CMK-3 catalyst.
